# No association between the rs10503253 polymorphism in the *CSMD1* gene and schizophrenia in a Han Chinese population

**DOI:** 10.1186/s12888-016-0923-5

**Published:** 2016-07-04

**Authors:** Yansong Liu, Zaohuo Cheng, Jun Wang, Chunhui Jin, Jianmin Yuan, Guoqiang Wang, Fuquan Zhang, Xudong Zhao

**Affiliations:** Department of Psychosomatic Medicine, Shanghai East Hospital, Tongji University School of Medicine, Shanghai, 200092 China; Wuxi Mental Health Center, Wuxi, 214151 Jiangsu Province China

**Keywords:** Schizophrenia, *CSMD1*, rs10503253

## Abstract

**Background:**

Schizophrenia (SCZ) is a complex, heritable, and devastating psychiatric disorder. Recent genome-wide association studies have identified a single-nucleotide polymorphism (SNP; rs10503253) in the CUB and SUSHI multiple domains 1 (*CSMD1*) gene as a risk factor for SCZ. In this study, we investigated whether the rs10503253 in *CSMD1* contributes to the risk of SCZ in a Han Chinese population.

**Methods:**

We conducted a case-control study in a population from eastern China, involving 1378 SCZ patients and 1091 unrelated healthy controls, using the ligase detection reaction–polymerase chain reaction method to genotype the rs10503253 polymorphism in the *CSMD1* gene.

**Results:**

No significant association was found between the SCZ patients and controls for any allele or genotype frequency of the SNP rs10503253 (all *P* > 0.05).

**Conclusions:**

Our findings do not support an association between *CSMD1* rs10503253 and SCZ in a Han Chinese population.

## Background

Schizophrenia (SCZ) is a common and heritable psychiatric disorder that affects approximately 1 % of the general population worldwide, characterized by hallucinations, delusions and cognitive deficits, and incompatibility of mental activity and environment. Although genetic factors account for more than 80 % of the etiology and heritability of SCZ, the exact etiology and genetic mechanism of the disease are still unknown [1, 2].

The CUB and Sushi multiple domains-1 (*CSMD1*) gene, located on 8p23.2, is a complement control-related protein, and *CSMD1* is highly expressed in the central nervous system (CNS) [3]. Although the exact functions of this gene remain unclear, it has been reported that *CSMD1* may play an important role in modulating the ratio between dopamine and serotonin metabolites in CNS [4]. Recently, a single-nucleotide polymorphism (SNP) of *CSMD1*, rs10503253, has been identified by a large-scale genome-wide association study (GWAS) in samples of European ancestry as a risk genetic variant for SCZ [5]. Subsequent studies have replicated the association of rs10503253 with SCZ in subjects of European ancestry [6, 7]. This association was recently confirmed in an enlarged GWAS [8] by the group [5].

The *CSMD1* gene has been validated as a target of miR-137 [9], which is involved in regulation adult neurogenesis [10] and neuron maturation [11], and is related to the pathogenesis of SCZ. In addition, several studies have revealed that the A allele of rs10503253 is associated with cognitive dysfunction, which has been documented as a primary and core deficit of SCZ [7, 12]. Therefore, *CSMD1* is considered to be an important susceptibility gene for SCZ.

Although several studies supported the association of *CSMD1* rs10503253 with SCZ, Ohi et al. [13] failed to replicate this result in samples from Japanese descent. Given that different ethnic populations may exhibit genetic heterogeneity of SCZ and only several genes have been consistently associated with SCZ across multiple studies in different genetic populations, it is necessary to investigate the possible relationship between this genetic locus and SCZ among other independent populations.

To further evaluate the association of *CSMD1* rs10503253 with SCZ, we conducted a case-control study in an independent Han Chinese population from Jiangsu province.

## Methods

### Subject recruitment

The study sample included 1378 patients with SCZ (854 males and 524 females; mean age = 45.92 years at recruitment, standard deviation (SD) = 11.51) and 1091 matched unrelated healthy controls (607 males and 484 females; mean age = 45.01 years at recruitment, SD = 10.31), drawn from a population of people of Han descent (Table 1).

**Table 1:** Demographic characteristics of the study subjects

Group	Case (n, %)	Control (n, %)
Sex		
Total	1378	1091
M	854 (62)	607 (56)
F	524 (38)	484 (44)
Age		
Range	14–76	16–77
Mean	45.92 ± 11.51	45.01 ± 10.31
14–19	10 (1)	4 (0)
20–29	141 (10)	97 (9)
30–39	201 (15)	174 (16)
40–49	489 (35)	455 (42)
50–59	375 (27)	275 (25)
60–69	151 (11)	80 (7)
70–76	11 (1)	6 (1)

*M* Male, *F* Female

For the patient group, the diagnosis of SCZ was confirmed by at least two experienced psychiatrists according to the Structured Clinical Interview for DSM-IV (SCID-I) and the Diagnostic and Statistical Manual of Mental Disorders (DSM-IV) criteria for SCZ. Exclusion criteria included the presence of other mental disorders, neurodevelopmental disorders or significant organic brain injury, epilepsy, and alcohol addiction or substance abuse. For the selection of control subjects, the Structured Clinical Interview for DSM-IV and Non-patients edition (SCID-NP) were used to interview members of an unrelated healthy general population, and those who met any psychiatric diagnosis within the SCID-I Axis I were excluded. Subjects with any family history of psychiatric disease among first-degree relatives were also excluded. The control subjects lived in the same geographical area as the patients, and were group matched to the patients by age, gender, and ethnicity. Any subject not born in Jiangsu was excluded, based on a self-reported questionnaire regarding the ancestral and origin birth places.

The clinical research was in compliance with the World Medical Association Declaration of Helsinki—Ethical Principles for Medical Research Involving Human Subjects. This study was approved by the Ethics Committees of the Wuxi Health Mental Center, and signed informed consent was obtained from all participants, or from his/her guardians when the participant had no capacity to consent. Healthy subjects were recruited by advertisement. Before being enrolled in the present study, each subject was also required to sign a consent form.

### DNA extraction and SNP genotyping

Peripheral blood samples were collected from all subjects using K_2_EDTA tubes. Genomic DNA was extracted from 150 μl of peripheral blood using a blood genotyping DNA extraction kit (Tiangen Biotech, Beijing, China). DNA samples were then stored at −80 °C for genotype analysis.

SNP genotyping was performed using the ligase detection reaction–polymerase chain reaction (LDR-PCR) method [14, 15] by the Shanghai Biowing Applied Biotechnology Co., Ltd. (www.biowing.com.cn). Genomic DNA extracted from the clinical samples was first subjected to multiplex PCR to obtain a PCR product including SNPs. The PCR primers for SNP rs10503253 were 5’-CATAAGTTTATATTTCTCAC-3’ (forward) and 5’-CTGTAGCAGGTTCAACAGAC-3’ (reverse). This PCR product and the LDR probes were then subjected to a multiplex LDR reaction, with a DNA sequencer used to detect the products.

### Statistical analysis

All statistical analyses were performed using the online software platform SHEsis (http://analysis.bio-x.cn/myAnalysis.php) [16], including association studies, Hardy–Weinberg equilibrium (HWE) tests, and the calculation of genotype and allele frequencies between patients with SCZ and healthy controls. The power analysis for our sample size was performed using the G*power analysis program [17] based on Cohen’s method [18]. *P* values of less than 0.05 were considered statistically significant.

## Results

Data from 1378 patients with SCZ and 1091 unrelated healthy controls were analyzed. HWE tests indicated that the genotype frequency distribution of rs10503253 did not significantly deviate from HWE (*P* = 0.15 for cases; *P* = 0.35 for controls). The total genotyping rate in all individuals was 99.51 %. In addition, we calculated the power of this study using the G*power program described by Li et al [19]. The results showed that our sample size revealed 99.87 % power to detect a significant association (α < 0.05) when an effect size index of 0.1 (corresponding to a weak gene effect) was used. When an effect size index of 0.2, which corresponds to a weak to moderate gene effect was used, the sample size showed 100 % power in detecting a significant (a < 0.05) allele and genotype association.

The results of our association study suggested that the A allele of SNP rs10503253 within *CSMD1* was not significantly associated with SCZ risk (*P* = 0.26, *OR* = 1.07, 95 % *CI*: 0.95–1.21) in a Han Chinese population sample (see Table 2). Furthermore, stratification analysis by gender did not show any positive association between SCZ and the A allele of SNP rs10503253. A detailed summary is shown in Table 3. The statistical results suggest that the A allele of SNP rs10503253 in *CSMD1* might not be a risk locus for SCZ susceptibility in Han Chinese populations.

**Table 2:** Frequencies of alleles and genotypes for rs10503253 in SCZ patients and controls

Group	n	Allele		*P*	OR (95 % CI)	genotype			*P*	HWE
		A (n, %)	C (n, %)			A/A (n, %)	A/C (n, %)	C/C (n, %)		
Case	1378	861 (31)	1895 (69)	0.26	1.07 (0.95–1.21)	123 (9)	615 (45)	640 (46)	0.50	0.15
Control	1091	649 (30)	1533 (70)			90 (8)	469 (43)	532 (49)		0.35

*OR* Odds Ratio, *95 *% *CI* 95 % Confidence Interval, *HWE* Hardy–Weinberg equilibrium

**Table 3:** Frequencies of alleles and genotypes for rs10503253 in gender groups

Group	*n*	Allele		*P*	OR (95 % CI)	genotype			*P*
		A (n, %)	C (n, %)			A/A (n, %)	A/C (n, %)	C/C (n, %)	
M-case	854	520 (30)	1188 (70)	0.90	1.01 (0.86–1.19)	74 (9)	372 (43)	408 (48)	0.76
M-control	607	367 (30)	847 (70)			47 (8)	273 (45)	287 (47)	
F-case	524	341 (33)	707 (67)	0.10	1.17 (0.97–1.42)	49 (10)	243 (46)	232 (44)	0.12
F-control	484	282 (29)	686 (71)			43 (9)	196 (40)	245 (51)	

*M* Male, *F* Female

## Discussion

Previous studies have indicated that the activity of complement components (including complement component C3) is tightly controlled in the brain [20], and deregulation of complement has been found to be involved in psychiatric disorders such as SCZ [21, 22]. *CSMD1* is a complement control-related protein and plays a role in inhibition of complement C3 activation [23, 24]. In addition, it has been reported that *CSMD1* is under the control of miR-137 [9], which is implicated in the regulation of adult neurogenesis [10] and neuron maturation [11], and is therefore related to the pathogenesis of SCZ. Furthermore, the *CSMD1* gene has been reported to be associated with cognitive dysfunction, which has been recognized as a primary deficit in SCZ [7, 12]. These results suggest that the *CSMD1* gene could be an important factor for SCZ.

Recent GWASs have identified the A allele of SNP rs10503253 as a risk allele for SCZ [5–8]. However, Ohi et al. [13] failed to replicate the association between rs10503253 and SCZ. In our dataset, we also did not detect the association of rs10503253 with SCZ in a Han Chinese population. This inconsistency might result from the different genetic backgrounds. In the current study, the Han Chinese samples were all recruited from the Jiangsu province of China, the participants in the study by Ohi et al. [13] were people of Japanese descent, while the study samples in the studies by Ripke et al. [5] and Cross-Disorder Group of the Psychiatric Genomics Consortium [6] were all individuals of European ancestry. Furthermore, the samples in the study by Ripke et al. [8] were cohorts of admixed ethnicities from European and East Asian ancestry. The A allele frequencies of the rs10503253 SNP of *CSMD1* in healthy controls from our study and the studies by Ohi et al. [13], Ripke et al. [5], Cross-Disorder Group of the Psychiatric Genomics Consortium [6] and Ripke et al. [8] were 0.30, 0.32, 0.19, 0.20 and 0.22, respectively. The differences in the *CSMD1* polymorphism profiles suggest that populations from different ethnicities might exhibit genetic heterogeneity of SCZ. Additionally, sampling errors caused by differences in clinical diagnosis of SCZ might also contribute to the inconsistency.

There are some limitations to the interpretation of our results. First, in this study we only investigated the SNP rs10503252 in the *CSMD1* gene. Other SNPs across the *CSMD1* gene may be linked with SCZ and cause alterations in Han Chinese populations. Second, the Han Chinese participants in this study were all recruited from Jiangsu Province, and Han Chinese populations from different geographical areas might also exhibit genetic heterogeneity of SCZ. It is therefore unlikely that our findings can be extended to all people of Han descent. Third, the sample size of our study is still relatively small compared with current large-scale genetic studies [5, 6, 8], and the association of *CSMD1* with SCZ needs to be tested in more samples.

## Conclusions

In summary, this case-control study in a Han Chinese population did not support the association of *CSMD1* rs10503252 with SCZ risk, as reported in earlier GWASs studies. We hope that this finding contributes to a better understanding of the relationship between *CSMD1* genetic variants and SCZ pathogenesis. However, other SNPs within *CSMD1* are still possible risk SNPs for SCZ susceptibility, and further research could focus on finding other potential risk variants within *CSMD1* for SCZ.

## Abbreviations

CI, confidence interval; *CSMD1*, CUB and SUSHI multiple domains 1; DSM-IV, Diagnostic and Statistical Manual of Mental Disorders, 4th edition; F, female; GWASs, genome-wide association studies; HWE, Hardy–Weinberg equilibrium; M, male; OR, odds ratio; PCR, polymerase chain reaction; SCZ, schizophrenia; SNP, single-nucleotide polymorphism
